# Nanoindentation of Bi_2_Se_3_ Thin Films

**DOI:** 10.3390/mi9100518

**Published:** 2018-10-14

**Authors:** Hong-Da Lai, Sheng-Rui Jian, Le Thi Cam Tuyen, Phuoc Huu Le, Chih-Wei Luo, Jenh-Yih Juang

**Affiliations:** 1 Department of Materials Science and Engineering, I-Shou University, Kaohsiung 84001, Taiwan; laihongdar95@gmail.com; 2 Department of Materials Science and Engineering, National Chiao Tung University, Hsinchu 30010, Taiwan; ltctuyen89@gmail.com; 3 Theoretical Physics Research Group, Advanced Institute of Materials Science, Ton Duc Thang University, Ho Chi Minh City 700000, Vietnam; 4 Faculty of Applied Sciences, Ton Duc Thang University, Ho Chi Minh City 700000, Vietnam; 5 Department of Electrophysics, National Chiao Tung University, Hsinchu 30010, Taiwan; cwluo@mail.nctu.edu.tw (C.-W.L.); jyjuang@g2.nctu.edu.tw (J.-Y.J.)

**Keywords:** Bi_2_Se_3_ thin films, nanoindentation, hardness, pop-in

## Abstract

The nanomechanical properties and nanoindentation responses of bismuth selenide (Bi_2_Se_3_) thin films are investigated in this study. The Bi_2_Se_3_ thin films are deposited on *c*-plane sapphire substrates using pulsed laser deposition. The microstructural properties of Bi_2_Se_3_ thin films are analyzed by means of X-ray diffraction (XRD). The XRD results indicated that Bi_2_Se_3_ thin films are exhibited the hexagonal crystal structure with a *c*-axis preferred growth orientation. Nanoindentation results showed the multiple “pop-ins” displayed in the loading segments of the load-displacement curves, suggesting that the deformation mechanisms in the hexagonal-structured Bi_2_Se_3_ films might have been governed by the nucleation and propagation of dislocations. Further, an energetic estimation of nanoindentation-induced dislocation associated with the observed pop-in effects was made using the classical dislocation theory.

## 1. Introduction

Recently, topological insulators (TIs) have attracted enormous research attention owing to their intriguing fundamental physical properties, such as their conduction mechanisms [1,2], as well as their potential applications in the emergent fields of spintronics [3], optoelectronics [4] and quantum computation [5]. Among various TI materials based on Bi compounds [6,7], bismuth selenide (Bi_2_Se_3_) is one of the most popular representative candidates in three-dimensional TIs [7,8] suitable for electronic applications, because of its large bulk energy gap of 0.3 eV and a single Dirac cone in the Brillouin zone [1,7]. In addition, Bi_2_Se_3_ also exhibits excellent thermoelectric properties at roomtemperature [9] and low-temperature regime [10]. For the fundamental study and device application, it is essential to grow Bi_2_Se_3_ thin films with high-quality and desired mechanical properties [11,12].

Epitaxial Bi_2_Se_3_ thin films have been successfully prepared by molecular beam epitaxy (MBE) [13,14,15,16]. Compared to MBE deposition, pulsed laser deposition (PLD) offers advantages such as a higher instantaneous deposition rate, relatively high reproducibility, and low costs. Thus, PLD has become one of the most widely used deposition techniques for growing thin films containing multi-elements. Both epitaxial and polycrystalline Bi_2_Se_3_ thin films have been successfully prepared by PLD [9,17,18,19,20]. In particular, PLD-grown Bi_2_Se_3_ thin films on InP (111) substrate presented triangular pyramids with step-and-terrace structures and growth along the [0001] direction [17]. Though lattice misfit over 13%, the Bi_2_Se_3_ films were epitaxially grown on Al_2_O_3_ (0001) with in-plane the relationship of (0001) Bi_2_Se_3_ ||(0001) Al_2_O_3_ and $\left[2\bar{1}\bar{1}0\right]$ Bi_2_Se_3_ ||$\left[2\bar{1}\bar{1}0\right]$ Al_2_O_3_ or $\left[2\bar{1}\bar{1}0\right]$ Bi_2_Se_3_ ||$\left[11\bar{2}0\right]$ Al_2_O_3_ [19]. Meanwhile, the Bi_2_Se_3_ films prepared by metal organic chemical vapor deposition and thermal evaporation exhibited polycrystalline morphologies and c-axis preferred oriented structures [21,22]. In this study, PLD technique is adopted to grow textured Bi_2_Se_3_/Al_2_O_3_ (0001) thin films and study their nanomechanical properties.

The mechanical properties of thin films in nanometer-scale are of great interest since they can be significantly different from their bulk counterparts. Especially, when thin films are used as structural/functional elements of certain nanodevices, robustness to stringent mechanical impacts arising from various fabrication processes is also of pivotal importance. Thus, studies on the correlations between the microstructural and mechanical properties of thin films are indispensable. Nanoindentation has been widely used as a powerful depth-sensing probe for measuring the primary mechanical property parameters, such as hardness and elastic modulus, as well as in revealing the plastic deformation behaviors and mechanisms of various nanoscaled materials [23,24,25,26], thin films [27,28,29,30,31] and single-crystal materials [32,33]. Herein, we report the nanomechanical properties of Bi_2_Se_3_ thin films deposited on *c*-plane sapphire substrates by PLD using nanoindentation with the aid of the continuous contact stiffness (CSM) mode. In addition to obtaining the characteristic nanomechanical properties of Bi_2_Se_3_ thin films, we also performed detailed analyses on the first pop-in event displayed on the load-displacement curves of nanoindentation to elucidate the underlying plastic deformation mechanisms and the associated dislocation physics [34,35,36,37].

## 2. Materials and Methods

The Bi_2_Se_3_ thin films investigated in the present study were deposited on Al_2_O_3_ (0001) substrates by using PLD at a substrate temperature of 300 °C with a helium ambient pressure of 220 mTorr. In particular, in order to obtaining near stoichiometric films at the relatively high substrate temperature of 300 °C, the Se-rich target with a nominal composition of Bi_2_Se_8_ was used. For the PLD process, ultraviolet (UV) pulses (20-ns duration) from a KrF excimer laser (λ = 248 nm, repetition: 5 Hz) were focused on a polycrystalline Bi_2_Se_8_ target at a fluence of 6.25 J/cm^2^ and a target-to-substrate distance of 40 mm. The deposition time was 20 min, which resulted in an average Bi_2_Se_3_ film thickness of approximately 360 nm (the growth rate of approximately 0.6 Å/pulse).

The crystalline structure of the obtained Bi_2_Se_3_ thin films was examined by X-ray diffraction (XRD; Bruker D8, Bruker, Billerica, MA, USA) using theCuKα radiation, λ = 1.54 Å. The surface morphology and film compositions were analyzed by a field emission scanning electron microscopy (FESEM; JEOL JSM-6500, JEOL, Pleasanton, CA, USA) and an Oxford energy-dispersive X-ray spectroscopy (EDS) attached to the SEM instrument, respectively. The analyses were conducted using an accelerating voltage of 15 kV, with the dead time of 22–30% and collection time of 60 s, respectively.

The nanoindentation tests were carried out at a Nanoindenter MTS NanoXP^®^ system (MTS Cooperation, Nano Instruments Innovation Center, Oak Ridge, TN, USA). A three-sided pyramidal Berkovich-type diamond indenter tip with radius of curvature of 50 nm was used for all indentation measurements. The mechanical properties of Bi_2_Se_3_ thin films were measured by nanoindentation with the continuous contact stiffness (CSM) mode [38]. The indenter was loaded and unloaded three times to ensure that the tip was properly in contact with the material surface, and that any parasitic phenomenon was released from the measurements. Then, the indenter was loaded for the fourth and final time at a strain rate of 0.05 s^−1^, with a 5 s holding period inserted at the peak load in order to avoid the influence of creep on unloading characteristics, which were used to compute the mechanical properties of Bi_2_Se_3_ thin films. Finally, the indenter was withdrawn with the same strain rate until 10% of the peak load was reached. At least 20 indents were performed. We also followed the analytic method proposed by Oliver and Pharr [39] to determine the hardness and Young’s modulus of Bi_2_Se_3_ thin films. In order to investigate the cracking phenomenon, cyclic nanoindentation tests were also performed. For the first cycle, the indenter was loaded to some chosen load and then unloaded by 90% of the previous load. It then was reloaded to a larger chosen load and unloaded by 90% for the second cycle. Noticeably, in each cycle, the indenter was hold for 10 s at 10% of its previous maximum load for the thermal drift correction and for assuring unloading completion. The same loading/unloading rate of 10 mN/s was used. The thermal drift was kept below ±0.05 nm/s for all indentations.

## 3. Results

**Figure 1:**
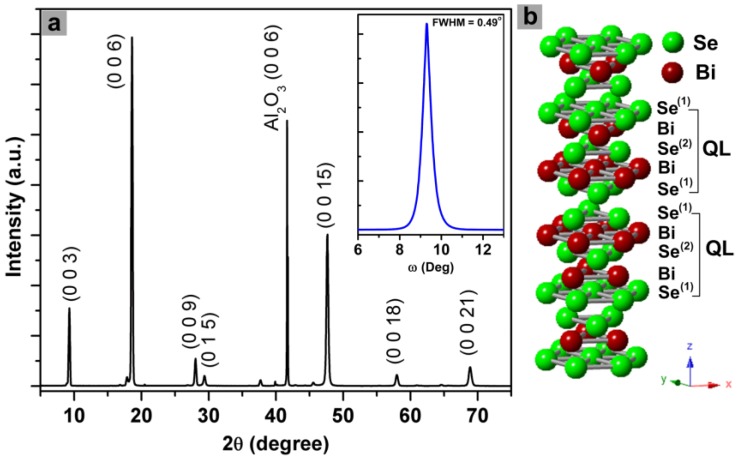
(**a**) X-ray diffraction (XRD) patterns of a bismuth selenide (Bi_2_Se_3_) thin film grown on *c*-plane sapphire using pulsed laser deposition (PLD). The inset in (a) shows the XRD rocking curve of (006) peak for the film. (**b**) Crystal structure of Bi_2_Se_3_ (QL is quintuple layer).

In Figure 1a, XRD patterns show the dominant (0 0 3*n*) diffraction peaks of Bi_2_Se_3_ films in addition to a minor Bi_2_Se_3_ (0 1 5) peak and a Al_2_O_3_ (0 0 6) peak of the substrate, indicating the film growth along the [0001] direction. This is due to the rhombohedral crystal structure of Bi_2_Se_3_ (space group $D_{3d}^{5}$($R\bar{3}m$)), in which a hexagonal primitive cell consists of three layers of –(Se^(1)^–Bi–Se^(2)^–Bi–Se^(1)^)–lamellae (called quintuple layers, QLs) stacking in sequence along the *c*-axis [15]. The interaction between the neighboring QLs is mainly the Se^(1)^–Se^(1)^ van der Waals bond (Figure 1b). The interlayer Se^(1)^–Se^(1)^ bonding not only is substantially weaker than the intralayer ionic-covalent bonds within individual QLs but also results in a lowest surface energy on the {001} planes, which leads to observed preferred (001)-oriented crystal growth behavior [9]. As shown the inset of Figure 1a, the full width half maximum (FWHM) of the (0 0 6) peak from the XRD rocking curve was found to be 0.49°, which suggests the presence of certain disorientation between grains (see also Figure 1b). This FWHM was comparable to that of Bi_2_Se_3_ film grown on Al_2_O_3_ by PLD [17]. Moreover, the in-plane orientation of the films were examined by XRD Ф-scan on {0 1 5} planes of the Bi_2_Se_3_ films at a tilt angle (χ) of 57.9°. The films did not show any diffraction peaks, indicating their in-plane polycrystalline characteristics.

**Figure 2:**
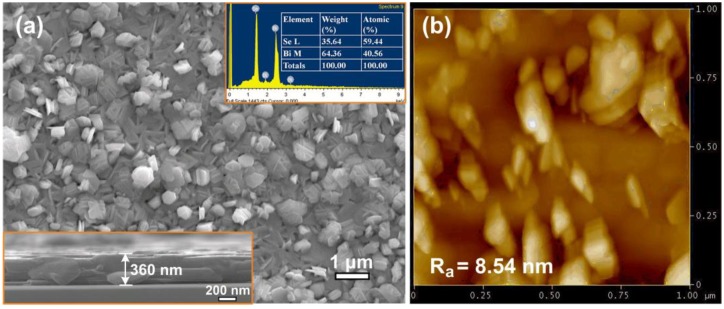
(**a**) A plane-view SEM image of the Bi_2_Se_3_ thin film deposited on *c*-plane sapphire. Lower inset: a cross-sectional SEM image of the film; upper inset: The energy-dispersive X-ray spectroscopy (EDS) spectra and relative compositions of the film. (**b**) AFM image of the film, *R_a_* is the center line average roughness.

Intriguingly, the films presented polycrystalline morphology with mutually crossed nanoplatelets (Figure 2a), which are somehow similar to those of Bi_2_Te_3_ grown by electrodeposition [40]. It has been proposed that the formation of mutually crossed Bi_2_Te_3_ nanoplatelets can be mainly attributed to the anisotropic bonding nature and growth facet planes with appropriate chemical stoichiometry [40]. This formation mechanism may be also prevailing in the present Bi_2_Se_3_ films due to the similar anisotropic bonding nature of Bi_2_Se_3_ and Bi_2_Te_3_. The film exhibited layered structure and uniform thickness of ~360 nm, as shown by the cross-sectional SEM image in Figure 2a. The upper inset of Figure 2a summarizes the EDS result of the film. Clearly, the film obtained stoichiometric composition of Bi_2_Se_3_ (i.e., 40.56 at.% Bi and 59.44 at.% Se). The surface roughness can be represented by center line average (*R_a_*), as shown by the AFM image in Figure 2b. The *R_a_* of the film was 8.54 nm.

**Figure 3:**
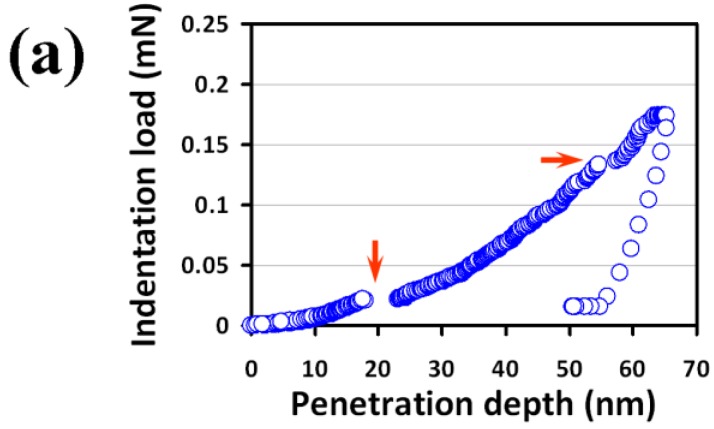

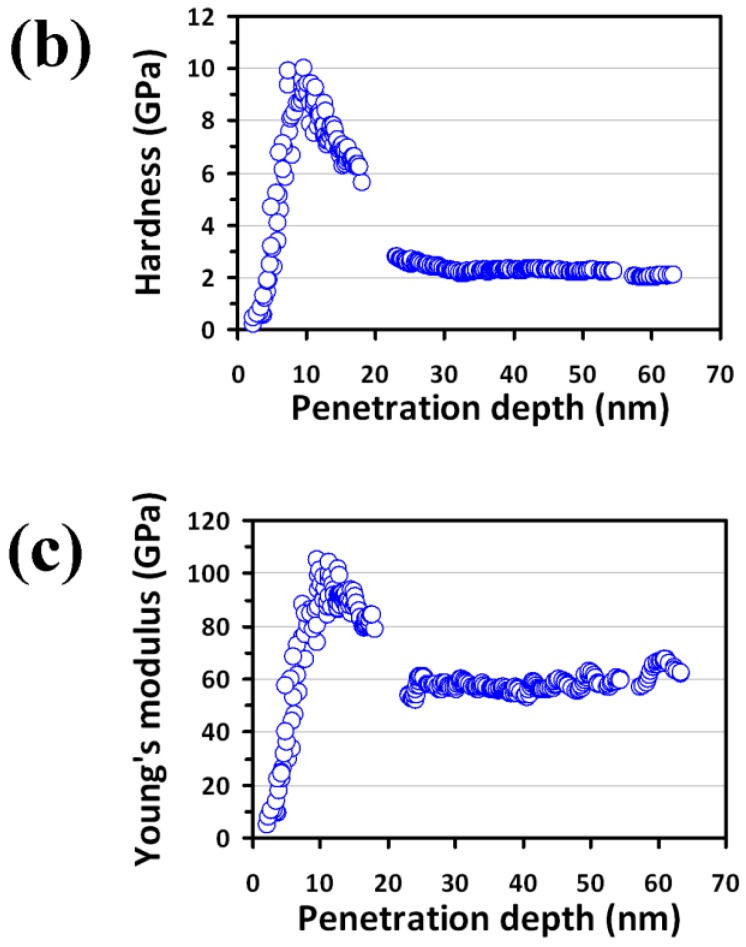
(**a**) A load-displacement curve showing the multiple “pop-ins” during loading part, (**b**) hardness-displacement curve and, (**c**) Young’s modulus-displacement curve are obtained from the nanoindentation continuous contact stiffness (CSM) results of Bi_2_Se_3_ thin film.

Figure 3a displays the typical load-displacement curve of the present Bi_2_Se_3_ films obtained by CSM. The corresponding indentation depth-dependent hardness and Young’s modulus are shown in Figure 3b,c, respectively. As is evident from Figure 3b, the indentation depth-dependent hardness of Bi_2_Se_3_ thin film can be roughly divided into two stages. Namely, the hardness, after reaching the maximum in the first 10 nm, precipitously decreases with further increasing indentation depth and eventually reaches a constant value at 2.1 ± 0.1 GPa after the first stage. It is noted that the present results are well within the 30% depth/thickness criterion for nanoindentation test suggested by Li et al. [23,41]. Thus, the effects arising from the substrate or film/substrate interface are excluded. In this respect, the “noisy” depth-dependent hardness, especially in the first stage, might be arisen from the extensive dislocation activities in this stress range. Similar tendency in the depth-dependent Young’s modulus is observed (Figure 3c), presumably due to the same mechanism. The Young’s modulus of the present Bi_2_Se_3_ thin film is 58.6 ± 4.1 GPa. It is interesting to note that both the hardness and Young’s modulus of present PLD-derived Bi_2_Se_3_ thin films are much larger than that of single-crystal Bi_2_Se_3_ reported by Gupta et al. [12], where the respective values of 85.09 MPa and 6.361 GPa were obtained. The reason for the apparent discrepancy is not clear at present. Nevertheless, in addition the apparent differences in microstructure, such as grain boundaries (see Figure 2a), we also note that the load and penetration depths carried out in Reference [12] were both much larger than that employed in the present study. Recently, it has been found in a hybrid double perovskite (MA)_2_AgBiBr_6_ that Young’s modulus decreased considerably with increasing indentation depth [42], which partially explains for the larger Young’s modulus in this study than that of in Reference [12].

**Figure 4:**
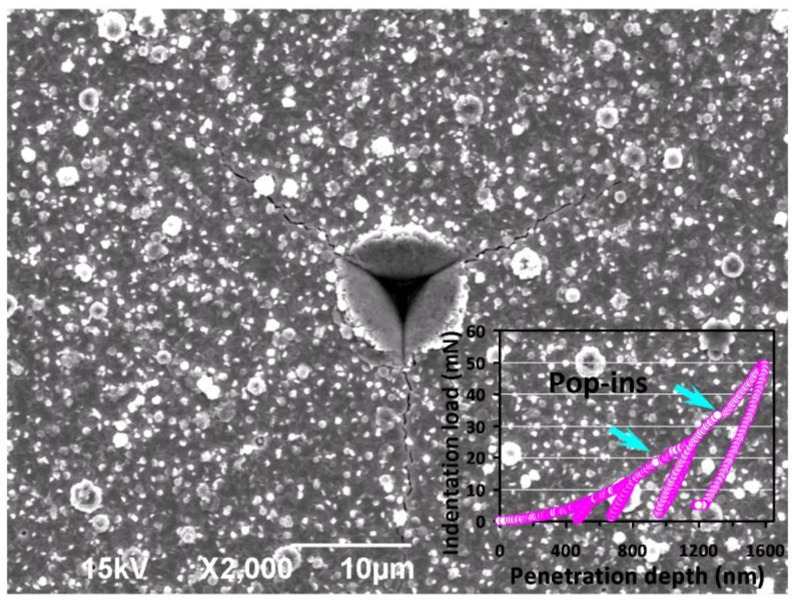
Nanoindented SEM micrograph of Bi_2_Se_3_ thin film showing cracks propagate along the corners and pile-up beside the edges of the Berkovich indent. The inset shows the cyclic load-displacement curve at a load of 50 mN. Notice that the multiple “pop-ins” is observable (indicated by the arrows) in loading segments.

From Figure 3a and the cyclic load-displacement curve in the inset of Figure 4, signatures of the multiple pop-ins are clearly observed in the loading part, as indicated by the arrows shown in both figures. It is noted that similar behaviors were also observed in nanoindented Bi_2_Se_3_ single crystals and was interpreted as being due to heterogeneous nucleation of dislocations beneath the indenter tip [12]. Since the multiple pop-ins is generally closely related to the sudden collective activities of dislocations [43] (such as dislocation generation or movement bursts), we believe that massive dislocation activities are the predominant deformation mechanism in this material, which, in fact, is also consistent with the conjectures of the resultant “noisy” features seen in the depth-dependent curves hardness and Young’s modulus described above.

It is also interesting to note that no “pop-out” event is observed in both the unloading curves displayed in Figure 3a and in the inset of Figure 4. Such pop-out behavior is often interpreted as a manifestation of indentation-induced phase transition (for example: nanoindentation-induced phase transformation of single-crystal Si [44]), which is not found in our case. However, as revealed by the SEM image shown in Figure 4, it is evident that significant cracks and pile-ups phenomena along the three corners and edges of the residual indent are also observable. The multiple pop-ins were observed in a large array of materials and were demonstrated to result mainly from massive nucleation and/or propagation of dislocations during loading [45], or micro-cracks initiated around the indentation tip [46]. Hence, it is clear that not only the first pop-in event may reflect the onset of plasticity due to the dislocation activities, but the cracking and pile-up event could also be dominated by the similar mechanism in the present Bi_2_Se_3_ thin films under nanoindentation. On the other hand, the pressure-induced structural phase transition in Bi_2_Se_3_ using high pressure Raman and XRD experiments [47] has evidenced that the magnitude of required pressure to induce phase transitions is significantly higher than the apparent room-temperature hardness of hexagonal Bi_2_Se_3_ thin film measured here. It is worthwhile mentioning that in many hexagonal structured materials, such as, sapphire [48] and GaN thin films [49,50,51,52], the primary nanoindentation-induced deformation mechanisms have been consistently identified to be the nucleation and propagation of dislocations. It is, thus, plausible to state that deformation behavior in the present Bi_2_Se_3_ thin films is most likely governed by the similar mechanisms.

Within the scenario of the dislocation nucleation and propagation, the first pop-in event appearing in the loading segment naturally reflects the onset of plasticity for Bi_2_Se_3_ thin film, which also provides prominent information about the critical shear stress (*τ*_max_) the energy associated with the nucleation of dislocation loops. Following the analytical model proposed by Johnson [53], *τ*_max_ can be related to the indentation load (*P_c_*), at which a discontinuity in the load-displacement curve takes place, through the following equation [53]:

(1) $$\tau_{\max}=\frac{0.31}{\pi }{\left[6P_{c}{\left(\frac{E_{r}}{R}\right)}^{2}\right]}^{1/3}\;$$

Here, *R* is the radius of indenter tip and *E_r_* is the effective elastic modulus, respectively. The maximum shear stress for Bi_2_Se_3_ thin films investigated in the present study is about 0.7 GPa. To the first approximation, the work done by this *τ*_max_ is mainly associated with the dislocations nucleated within the deformation region underneath the indenter tip. Assuming the nucleation is homogeneous during nanoindentation [34], then, according to the classical dislocation theory [54], the stress at which the first “pop-in” taking place and the energy “dissipated” in it can be regarded, respectively, as the shear stress required to initiate plastic deformation and the energy required for generating a dislocation loop to prevail the deformation. The free energy (*U*_F_) of a circular dislocation loop with radius *r* can be written as:

(2) $$U_{F}=\gamma_{\mathrm{dis}}2\pi r-\tau b\pi r^{2}\;$$

where $\gamma_{\mathrm{dis}}$ is the energy per unit length of the dislocation loop, *b* is the magnitude of Burgers vector (~0.4 nm) [55] and *τ* is the external shear stress acting on the dislocation loop, respectively. The first term on the right-hand side of Equation (2) describes the energy increased by forming a dislocation loop of radius *r* in an initially defect-free lattice. The second term is nothing but the strain energy released via work done by the applied stress (*τ*) to expand the dislocation loop over a displacement of one Burgers vector. The linear energy density ($\gamma_{\mathrm{dis}}$) for a dislocation is given by [54]:

(3) $$\gamma_{\mathrm{dis}}=\frac{G\;b^{2}}{8\pi }\left(\frac{2-v_{f}}{1-v_{f}}\right)\left[\ln\frac{4r}{r_{\mathrm{core}}}-2\right]$$

where *G*, *v_f_* and *r*_core_ are the shear modulus (≈24 GPa), the Poisson’s ratio (assumed to be 0.25) of Bi_2_Se_3_ thin film, and radius of dislocation core, respectively. Substituting Equation (1) and Equation (3) into Equation (2) gives:

(4) $$U_{F}=\frac{Gb^{2}r}{4}\left(\frac{2-v_{f}}{1-v_{f}}\right)\left(\ln\frac{4r}{r_{\mathrm{core}}}-2\right)-\pi br^{2}\tau_{c}\;$$

Here, *τ*_c_ is the resolved shear stress of *τ*_max_ on the active slip systems of the material and is usually taken as half value of *τ*_max_ [56]. Equation (4) clearly indicates that *U*_F_ contains terms with first and second power of *r*. Thus, there must exist a critical radius, *r*_c_, at which *U*_F_ of the system reaches a maximum value. When the radius of the dislocation loop exceeds *r*_c_, further expansion lowers *U*_F_, hence is thermodynamically favorable. In contrast, if *r* < *r*_c_, the loop would shrink to reduce the energy. Consequently, when the loading reaches to the “pop-in” point, homogeneous formation of circular dislocation loop becomes possible without thermal energy at *U*_F_ = 0 [57]. The condition (*U*_F_ = 0) allows *τ*_c_ to be determined from through Equation (2) and Equation (3), yielding $r_{c}=2\gamma_{\mathrm{dis}}/(b\tau_{\max})$. Since *τ*_c_ has a maximum value as $d\tau_{c}/dr=0$, one obtains: $r_{c}=(e^{3}r_{\mathrm{core}})/4$. The values of *r*_core_ and *r*_c_ for the present Bi_2_Se_3_ thin films were calculated to be 1.08 nm and 5.4 nm, respectively.

**Figure 5:**
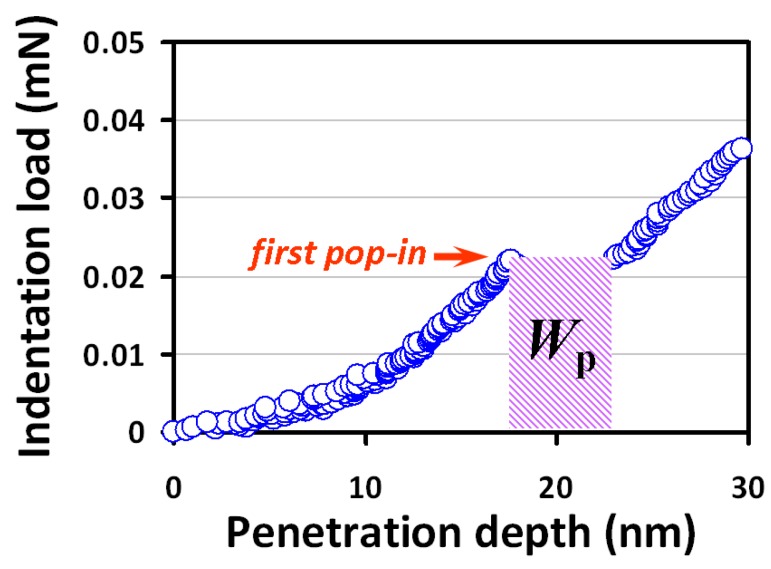
The corresponding first pop-in event from Figure 3a is zoomed in to depict the plastic strain work, *W*_p_, which is approximated as the product of critical loading and the sudden incremental displacement indicated by the shaded area.

By assuming that the nucleation of dislocation loops is entirely responsible for the indentation-induced plastic deformation and no thermal effect is involved, one can further estimate the number of dislocation loops formed during the first “pop-in” event by using the associated work-done (*W*_p_). As depicted in Figure 5, the estimated *W*_p_ is ~0.11 × 10^−12^ Nm, suggesting that ~8 × 10^3^ dislocation loops with critical diameter might have been formed. Although the estimated number is relatively low compared to that of typical polycrystalline thin films (~10^6^ cm^−2^) [58], it is, nevertheless, consistent with the scenario that the “pop-in” is induced by massive homogeneous dislocation nucleation, instead of by the activated collective motion of pre-existing grown-in dislocations [34]. Alternatively, one can take the total dissipation energy as the energy to estimate the number of dislocations with critical radius being generated during entire nanoindentation practice. In that case, as high as ~3 × 10^5^ dislocation loops may be formed during nanoindentation. This number, albeit not entirely realistic, may be considered as the upper limit within the context of dislocation dominant deformation mechanism.

## 4. Conclusions

To sum up, XRD, SEM, AFM and nanoindentation techniques are used to investigate the microstructural and surface morphological features, as well as the nanomechanical properties of Bi_2_Se_3_ thin films. The results show that the Bi_2_Se_3_ thin films are polycrystalline with highly (00*l*)-orientation (texture films) and stoichiometric compositions. The hardness and Young’s modulus of Bi_2_Se_3_ thin film are obtained 2.1 ± 0.1 GPa and 58.6 ± 4.1 GPa, respectively. Similar to many hexagonal-structured semiconductors, the primary deformation mechanism for the present Bi_2_Se_3_ thin film is governed by nucleation and propagation of dislocations or the formation of cracking events. Preliminary energetic estimations indicated that the number of dislocation loops induced by nanoindentation to trigger the plastic deformation accounts for the first pop-in event was in the order of 10^3^ with a critical radius (*r_c_* ≈ 5.4 nm). Although the estimated dislocation density is relatively low compared to that of typical polycrystalline films, it is, nevertheless, in line with the scenario of homogeneous dislocation nucleation-induced first “pop-in” event.
